# Cholera

**Published:** 1885-09

**Authors:** 


### Book Reviews.

CHOLERA.—By Alfred Stillé, M. D., LL. D., Professor Emeritus of the Theory and Practice of Medicine in the University of Pennsylvania. Philadelphia: Lea, Brothers & Co. 1885. 8vo. pp. 164.

The author says in the preface that the "interest awakened by the possibility of the advent of cholera has led to the publication of the treatise." The book is written in a popular style, and gives a resumé of our knowledge, up to date, of this terrible scourge. The work opens with an epitome of the history of the disease, giving a map and the salient points connected with epidemics as far back as records go, up to the present time. Then follows successively the etiology, symptomatology, pathology, diagnosis, prognosis and treatment. The author does not accept the comma bacillus of Koch as the *causa morbis*, but holds that the essential cause of cholera is a "specific poison, which originates in Hindostan. . . . Regarding the form and nature of that poison little or nothing is definitely established." The chapter on prevention contains much valuable advice, and advocates a strict quarantine whenever an epidemic threatens. In the matter of treatment, the writer is conservative, and has little faith in the curative power of medicine in cases of true Asiatic cholera. The treatment advised is symptomatic, and it is claimed that "success depends upon prompt and early application." The book is eminently practical, and coming from the pen of one having a ripe experience, it carries the weight it deserves. The remark at the close of the book, concerning the work of Dr. Ferran, seems almost prophetic.

